# Communication‐related assessments in an Angelman syndrome mouse model

**DOI:** 10.1002/brb3.1937

**Published:** 2020-11-05

**Authors:** Peter A. Perrino, Stormy J. Chamberlain, Inge‐Marie Eigsti, Roslyn Holly Fitch

**Affiliations:** ^1^ Department of Psychological Science/Behavioral Neuroscience University of Connecticut Storrs CT USA; ^2^ Department of Genetics and Genome Sciences University of Connecticut Health Center Farmington CT USA; ^3^ Department of Psychological Science/Clinical Psychology University of Connecticut Storrs CT USA

**Keywords:** angelman syndrome, auditory processing, motor system, neurodevelopment, UBE3A, ultrasonic vocalizations

## Abstract

**Introduction:**

Angelman syndrome (AS) is a neurodevelopmental disorder characterized by motor deficits, seizures, some autistic‐like behaviors, and severe impairment of speech. A dysfunction of the maternally imprinted *UBE3A* gene, coupled with a functional yet silenced paternal copy, results in AS. Although studies of transgenic mouse models have revealed a great deal about neural populations and rescue timeframes for specific features of AS, these studies have largely failed to examine intermediate phenotypes that contribute to the profound communicative disabilities associated with AS.

**Methods:**

Here, we use a variety of tasks, including assessments of rapid auditory processing and social communication. Expressive vocalizations were directly assessed and correlated against other core behavioral measures (motor, social, acoustic perception) to model putative influences on communication.

**Results:**

AS mice displayed the characteristic phenotypes associated with Angelman syndrome (i.e., social and motor deficits), as well as marginal enhancements in rapid auditory processing ability. Our characterization of adult ultrasonic vocalizations further showed that AS mice produce fewer vocalizations and vocalized for a shorter amount of time when compared to controls. Additionally, a strong correlation between motor indices and ultrasonic vocalization output was shown, suggesting that the motor impairments in AS may contribute heavily to communication impairments.

**Conclusion:**

In summary, the combination of motor deficits, social impairment, marginal rapid auditory enhancements, and altered ultrasonic vocalizations reported in a mouse model of AS clearly parallel the human symptoms of the disorder. This mouse model offers a novel route to interrogate the underlying genetic, physiologic, and behavioral influences on the under‐studied topic of impaired communication in AS.

## Graphical abstract

AS model mice display rapid auditory processing impairments and produce atypical ultrasonic vocalizations. Findings suggest that language‐rescue efforts in AS populations should focus on treatments and timeframes that can rescue motor ability.

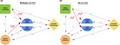

## 1 INTRODUCTION

Angelman Syndrome (AS) affects 1 in 15,000 live births (Mertz et al., 2013; Õiglane‐Shlik et al., 2006) and is characterized by developmental delays, epilepsy, difficulties with motor control, microcephaly, abnormal laughter, and social behavior, and altered sleep patterns (Mabb *et al.,* 2011; Williams et al., 2006). AS symptoms are present during the first year of life (Fryburg et al., 1991), and therefore AS is classified as a neurodevelopmental disorder. AS is further characterized by atypical social communication, which is also a core symptom of autism spectrum disorders (ASD; Williams et al., 2006). Less is known about the social communication symptom domain of AS compared with other symptoms, particularly as it relates to the genetic underpinnings of the disorder. Identification of genetic influences on core behavioral systems that influence social communication in AS is the focus of the current study.

AS is caused by a genetic mutation or deletion in the maternally inherited *UBE3A* allele (15q11.2; Kishino et al., 1997; Margolis et al., 2015). Since the paternal copy of the gene is silenced in neurons, mutation or deletion of the maternal allele results in little to no *UBE3A* gene product in neurons. The *UBE3A* gene encodes a protein product also known as E6‐associated protein (E6AP), which is a HECT domain‐containing E3 ubiquitin ligase (Huibregtse et al., 1993; Mabb & Ehlers, 2010). This protein first received attention for its role in the ubiquitination and degradation of the tumor repressor p53 (Scheffner et al., 1993). Mutations in the HECT domain that primarily affect the ubiquitin ligase activity of UBE3A cause AS (Cooper et al., 2004; Nawaz et al., 1999), strongly suggesting that loss of ubiquitin ligase function from UBE3A has a serious impact on neurodevelopment. Importance of UBE3A in neurodevelopment is further demonstrated by a de novo missense mutation that disrupts a PKA phosphorylation site on the E3 ligase, upregulating UBE3A protein, with enhanced enzyme activity and increased dendritic spine growth. This increase in *UBE3A* activity and altered synaptic function may contribute to the pathogenesis of ASD (Jason et al., 2015).

Mouse models of AS have been developed to further understand the neuroscience of the disorder. In a seminal original paper on the model, Jiang et al., (1998) reported that mice with a heterozygous maternal knockout of the rodent homolog *Ube3a* (denoted as *Ube3a^m−/p+^* or more simply, *Ube3a* AS) displayed characteristic symptoms of AS, including motor dysfunction and seizures, context‐dependent learning impairments, deficits in hippocampal long‐term potentiation, and increased cytoplasmic p53 in Purkinje cells and hippocampal pyramidal neurons. Subsequently, Yashiro et al. (2009) and Sato & Stryker (2011) used the model to show effects on experience‐dependent cortical plasticity during a developmental time period in which sensory input greatly impacts synaptic connectivity. Recent work from Judson et al., (2016) used conditional *Ube3a* mouse models to show that GABAergic UBE3A loss is the driving factor underlying increased circuit excitability, contributing in turn to the seizure and epileptic phenotype seen in individuals with AS.

The current study was designed to further explore the role of *UBE3A* in the language‐ and socio‐communicative‐related impairments of AS using a *Ube3a* mutant mouse model. Social communication is a core and highly impactful feature of AS, yet it has been particularly resistant to rodent study, and hence basic research approaches. This study has two primary components: first, the study replicated patterns of atypical behavior in AS mice on motor‐related tasks (Sonzogni et al., 2018) and social‐related tasks (Stoppel & Anderson, 2017). Secondly, it included novel communication‐specific measures of complex auditory processing and expressive communicative vocalization. These latter tasks were selected for their ability to detect subtle rapid changes in complex spectrotemporal acoustic stimuli (e.g., speech), a skill fundamental to language development (Benasich & Tallal, 2002; Farmer & Klein, 1995; Fitch et al., 2001; Hari & Kiesilä, 1996; Kraus et al., 1996). Indeed, other language‐impaired populations show difficulties with processing rapidly changing auditory information, regardless of whether it is speech‐ or non‐speech‐based (Cohen‐Mimran & Sapir, 2007; Tallal et al., 1993; Tallal & Newcombe, 1978; Tallal & Piercy, 1973; Vandermosten et al., 2011). Moreover, infant acoustic processing indices accurately predict long‐term language outcomes, thus emphasizing the importance of intact auditory processing for typical language development (Benasich et al., 2006; Benasich & Tallal, 2002). We also looked to extend evidence of anomalous communicative vocalizations in AS mice (Jiang et al., 2010; Mandel‐Brehm et al., 2015; Stoppel & Anderson, 2017), specifically by examining relationships between ultrasonic vocalizations (USVs) produced by adult AS model mice, and other core behavioral measures. Thus, we assessed whether general motor deficits, social behavioral anomalies, and/or low‐level acoustic processing anomalies may be statistically related within‐subjects to anomalous USV vocalization production. This statistical modeling could shed light on the neurologic underpinnings of communicative deficits in AS. The novel results of the current study point strongly to *motor anomalies* (rather than atypical social or receptive‐acoustic processing systems) as a major functional correlate of communicative impairments in AS.

## 2 METHODS AND MATERIALS

### 2.1 Subjects

Female *Ube3a^FLOX/p+^* embryos were obtained from Dr. Benjamin D. Philpot at UNC‐Chapel Hill (Chapel Hill, NC) (Judson et al., 2016) and were rederived on a C57BL/6J background strain at the Center for Mouse Genome Modification (CMGM) at UConn Health (Farmington, CT)—a C57 background strain was chosen due to reports of audiogenic seizures for *Ube3a* knockout mice on a 129 background strain. Audiogenic seizures did not occur in AS C57 mice at the age of 3 months (Born et al., 2017; Mandel‐Brehm et al., 2015). *Ube3a* expression in the *Ube3a^FLOX/p+^* mice have been extensively characterized by its developers using a variety of Cre‐drivers (Berrios et al., 2016; Bruinsma et al., 2015; Judson et al., 2016; McCoy et al., 2017; Sidorov et al., 2018). For this study, CMV‐Cre male mice (B6.C‐Tg(CMV‐cre)1Cgn/J; JAX stock #006054) were obtained from The Jackson Laboratory (Bar Harbor, ME) and crossed with female *Ube3a^FLOX/p+^* mice to generate an F2 generation, which consisted of 15 litters averaging 5 mice per litter. This resulted in female *Ube3a^m−/p+^* (AS) mice and female *Ube3a^m+/p+^*/ Cre^+^ (Control) mice, as well as *Ube3a^FLOX/p+^* and wild‐type males. Subjects were genotyped using the protocols outlined in Judson et al., 2016.

Twenty‐two female AS mice and 17 female Control mice (C57BL/6J background; littermates) were used for experimentation—only females were selected for experimentation due to previous work assessing communication in an adult AS mouse model (Stoppel & Anderson, 2017) as an effort to replicate findings. Subjects were given food and water *ad lib* and single‐housed after postnatal day (P) 30 in standard Plexiglass mouse cages. All mice were kept on a 12 hr:12 hr light/dark cycle with experimentation occurring during their light cycle. The subject's testing order was randomized prior to the start of experimentation—experimenters remained blind to genotype. All testing procedures occurred in compliance with National Institutes of Health and approved by the University of Connecticut's Institutional Animal Care and Use Committee (IACUC).

### 2.2 Rotarod (P50 ‐ 53)

Testing began at P58 with the Rotarod task—a task used to assess sensorimotor ability and motor learning. Subjects were placed on a cylindrical drum (6 cm in diameter; 7.5 cm in width) that rotated at an accelerating rate—4 rotations per minute (RPM) to 40 RPM over the span of 2 min. Over the course of three consecutive days, each subject completed four trials with an intertrial interval of 15 min. The latency to fall from the rotating cylindrical drum (distance to fall = 20 cm) was recorded and averaged across trials per day.

### 2.3 Modified Three‐Chamber Social Preference Task (P62)

Subjects were assessed on a modified Three‐Chamber Social task to evaluate general sociability by exploiting a mouse's typical preference for a conspecific (i.e., social stimulus) over an object (i.e., nonsocial stimulus). See Yang et al., (2011) for original methods. After completing a five‐minute habituation period, subjects were placed in the center of a three‐chambered testing apparatus (overall dimensions: 40.5 cm × 62 cm × 23 cm; each chamber: 40.5 cm × 20 cm × 23 cm) and allowed to freely explore all chambers for ten minutes. During this testing session, one chamber contained a caged, same‐sex Control mouse (“Novel Mouse”), while the other chamber, on the opposite side of the apparatus, contained a novel object composed of colored Legos in an identical cage (“Novel Object”) (cage dimensions: 7.5 cm in diameter × 8 cm tall). The middle chamber where the subject was originally place remained empty. Using video tracking software, TopScan LITE (Clever Sys Inc, Reston, VA), the percent time interacting with the conspecific, as well as number of entries, distance travelled, and speed of travel (velocity) within the Novel Mouse Chamber, Novel Object Chamber, and the combination of both Novel Mouse and Novel Object Chambers (“Novel + Object Chamber”), was recorded and analyzed for each subject.

### 2.4 Ultrasonic Vocalizations (USVs) (P66)

**Figure 1:**
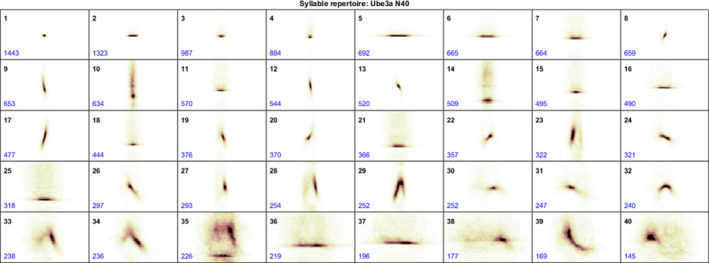
Syllable Repertoire generated by MUPET. Generated via MUPET analysis, forty unique syllables were assigned to one of ten possible categories (Heckman et al., 2016). Eight of the ten possible categories were observed in the syllable repertoire (Short (expected frequency range: 70 kHz; example: syllable #1), Flat (expected frequency range: 70 kHz; example, syllable #2), Down‐FM (expected frequency range: 80 – 60 kHz; example, syllable #9), Up‐FM (expected frequency range: 60 – 80 kHz example: syllable #20), Chevron (expected frequency range: 70 – 80 kHz; example: syllable #32), 2‐Frequency Step (expected frequency range: 60 – 80 kHz, example: syllable #35), Noisy (expected frequency range: 40 – 80 kHz example: syllable #36), and Complex (expected frequency range: >50 kHz example: syllable #39). The number located in the bottom‐left corner of each syllable indicates the number of times that syllable was produced across all subjects

Following Rotarod and Three‐Chamber Social testing, ultrasonic vocalizations were recorded and analyzed to assess socio‐communicative ability, using methods adapted from Ferhat et al., (2016). A single female subject was placed in a Plexiglass mouse tub (28 cm × 16.5 cm × 12 cm) and allowed to freely explore the chamber for twenty minutes. Following this habituation period, a second, same‐genotype female (i.e., “newcomer mouse”) was introduced and the two subjects were able to freely interact with one another. Under these conditions, the original female mouse will vocalize in the presence of the newcomer female mouse (Ferhat et al., 2016; Maggio and Whitney, 1985). Importantly, any vocalizations made by the newcomer mouse were balanced across genotype, so that total calls recorded came from with a Control or AS pair. During the ten‐minute interaction, a Brüel & Kjær Type 4954‐B microphone (Brüel & Kjær, Nærum, Denmark), recording at 192,000 Hz and connected to an RME Fireface UC audio interface (RME Audio, Haimhausen, Germany), was placed 5 cm above the top of the Plexiglass tub. Sound files (.*wav*) were recorded using DIGICheck 5.92 (RME Audio, Haimhausen, Germany) and analyzed in MATLAB (MathWorks) using MUPET (Mouse Ultrasonic Profile ExTraction) (Van Segbroeck et al., 2017). USVs that fell within the 35,000 Hz – 110,000 Hz range and had a duration between 8 ms and 200 ms were counted and termed a “syllable” (See Heckman et al., 2016 for review and definition & characteristics of each syllable category). Any USV that did not fall within this frequency or duration range, in addition to USVs that occurred less than 5 ms following a previous USV, were excluded from analyses. Following this inclusion criteria, a syllable repertoire was generated for all mice in the study that identified 40 unique syllables (not all mice made all syllables during their session; Figure 1). To further simplify analysis and reduce the number of statistical comparisons, each syllable was assigned to one of eight broader categories: Short (expected frequency range: 70 kHz; example: syllable #1), Flat (expected frequency range: 70 kHz; example, syllable #2), Down‐FM (expected frequency range: 80 – 60 kHz; example, syllable #9), Up‐FM (expected frequency range: 60 – 80 kHz example: syllable #20), Chevron (expected frequency range: 70 – 80 kHz; example: syllable #32), 2‐Frequency Step (expected frequency range: 60 – 80 kHz, example: syllable #35), Noisy (expected frequency range: 40 – 80 kHz example: syllable #36), and Complex (expected frequency range: >50 kHz example: syllable #39). Syllable duration (ms), syllable volume (dB), syllable pitch (kHz), the total number of syllables produced (syllable number), and total time spent vocalizing (s) were measured and analyzed.

### 2.5 Auditory Processing (P70)

#### 2.5.1 Modified prepulse inhibition paradigm

Subjects completed testing with a rapid auditory processing task that utilizes a modified prepulse inhibition paradigm (see Fitch et al., 2008 for review) where the subject's ability to suppress an acoustic startle response (ASR) is measured. Subjects were placed on a load‐cell platform (Med Associates, St. Albans, VT), covered with an open and opaque Plexiglass chamber (to prevent escape; 20.5 cm × 21.5 cm × 30.5 cm), and presented with auditory stimuli generated by RPvdsEx software and a RZ6 multifunction processor (Tucker Davis Technologies, Alachua, FL). The load‐cell platforms recorded the subject's motor reflex response to a 105dB, 50ms in duration, white noise burst ranging from 1,00Hz, ‐ 10,000Hz [known as the startle‐eliciting stimulus (SES)]. Information from the platforms was processed and recorded with a Biopac MP150 acquisition system and Acqknowledge 4.1 software (Biopac Systems, Goleta, CA).

The modified prepulse inhibition paradigm is used to assess the differences in ASR when the SES is presented with or without a preceding acoustic cue. The subject's cue detection/discrimination ability can be measured by analyzing differences in ASR between cued and uncued trials. In cued trials, an auditory stimulus (i.e., the cue) is presented 50ms before the SES and if the subject can detect the cue, their ASR will be reduced (attenuated) relative their ASR when the cue was not presented (i.e., uncued trial) or the cue was not detected. This phenomenon can be quantified by calculating an “attenuation score,” which compares the mean amplitude of the cued ASR to the mean amplitude of the uncued ASR:

$$\mathrm{Attenuation}\mathrm{Score}=\frac{\mathrm{mean}\mathrm{cued}\mathrm{ASR}}{\mathrm{mean}\mathrm{uncued}\mathrm{ASR}}x100$$

#### 2.5.2 Normal single tone (NST) (P70, P81)

Normal Single Tone (NST) was used to assess subject's baseline prepulse inhibition and general hearing ability. Against a silent background, subjects were asked to detect a 50ms auditory cue that consisted of a simple, 8,000Hz, pure tone (70dB) (NST 8kHz). 104 trials were used during the testing session, and on half of the trials, the cue was presented 50ms before the 105dB SES (i.e., cued trial) [*52 cued trials and 52 uncued trials; pseudorandomized, even trial distribution*]. Attenuation scores calculated from this task were used a covariate on subsequent auditory processing task as a measure to eliminate individualized differences in hearing ability and prepulse inhibition.

An ultrasonic version of this task was conducted following the completion of all nonultrasonic auditory tasks. The number of trials, intertrial interval durations, distribution of cued and uncued trials, and volumes for the cue and the SES were similar to that of NST 8kHz; however, the cue frequency was changed to 40,000Hz (NST 40kHz).

#### 2.5.3 Embedded tone 100 (P73 – 78)

The Embedded Tone (EBT) task tested the subject's the ability to detect a change in frequency within a constant, pure tone background (75 dB; 10,500 Hz). *Ube3a* AS and Control mice were subjected to 300 pseudorandom trials with the intertrial interval varying between 16 s – 24 s to ensure the subject was not able to predict the onset of the next trial. Cued trials contained a 5,600 Hz cue that occurred 100 ms before the 105 dB SES and varied in duration, ranging from 2 ms – 100 ms. Uncued trials contained a cue that occurred “0 ms” before the SES. EBT 100 was administered for five consecutive days.

#### 2.5.4 Single Arbitrary Waveform: Chevron (P82)

Subjects were required to detect a mouse ultrasonic vocalization—specifically, a chevron syllable from a wild‐type c57 mouse (44 ms in duration; amplitude ranged from 72,000 Hz – 85,000 Hz). Similar to NST, the cue was presented on half of the 104 trials 50 ms before the SES (pseudorandomly and evenly distributed) with ITIs ranging from 16s – 24s. Single Arbitrary Waveform (SAW): Chevron demands a higher level of processing and introduces a social‐context component to the discrimination requirement.

### 2.6 Statistical analysis

Behavioral data were subjected to statistical analysis by Task (Control, *n* = 17; AS, *n* = 22). For Rotarod, latency to fall from the rotating cylindrical drum was measured, and group differences were analyzed using a 2 (Genotype: Control and AS) × 3 (Day) repeated measures analysis of variance (ANOVA). For the Modified Three‐Chamber Social task, percent time interacting with the novel mouse was recorded, and group differences (Control and AS) were analyzed using a univariate ANOVA. For acoustic discrimination tasks, attenuation Scores were calculated for both versions of NST and SAW, and differences between Control and AS performance were analyzed using a univariate ANOVA. To assess Genotype differences on EBT 100, a 2 (Genotype: Control and AS) × 5 (Day) × 9 (Cue: 2 ms, 5 ms, 10 ms, 20 ms, 30 ms, 40 ms, 50 ms, 75 ms, and 100 ms) repeated measures ANOVA was used. For EBT 100 analysis, NST 8kHz was used as a covariate to account for any individual differences on hearing ability and prepulse inhibition. For USV recordings, syllable duration, syllable volume, syllable pitch, syllable number, and total time spent vocalizing were measured and analyzed (collapsed across category (overall) and by category) using a univariate ANOVA. Path models were assessed using linear bivariate correlation and linear multiple regression, with Bonferroni alpha‐correction for number of tests run; analyses were conducted with the help of UConn Statistical Consulting Center. All statistical analyses were conducted using SPSS 24 (IBM, Armonk, NY). The alpha criterion ranged from 0.05 to 0.10, two‐tailed, to capture significant results (i.e., ⍺ > 0.05) or to highlight nonsignificant trends (i.e., ⍺ > 0.10) (except where corrected for multiple tests). Data manipulation was performed using R version 3.5.1 (R Core Team, 2019). For power calculations, post hoc analyses were conducted to confirm appropriate Genotype size using G*Power 3.1 software (Faul et al., 2007) with α = 0.05 and power (1‐β) = 0.80.

## 3 RESULTS

Power analyses confirmed the Genotype size used was adequate to detect significance on tasks with moderate to large effect sizes. Future studies with a larger n per group may successfully delineate marginal effects such as the trend toward an auditory processing enhancement (i.e., Embedded Tone 0–100:10.5kHz and NST tasks) and specific characteristics of ultrasonic vocalizations (i.e., Syllable Pitch).

### 3.1 Motor Assessment (Rotarod & Modified Three‐Chamber Social Task)

**Figure 2:**
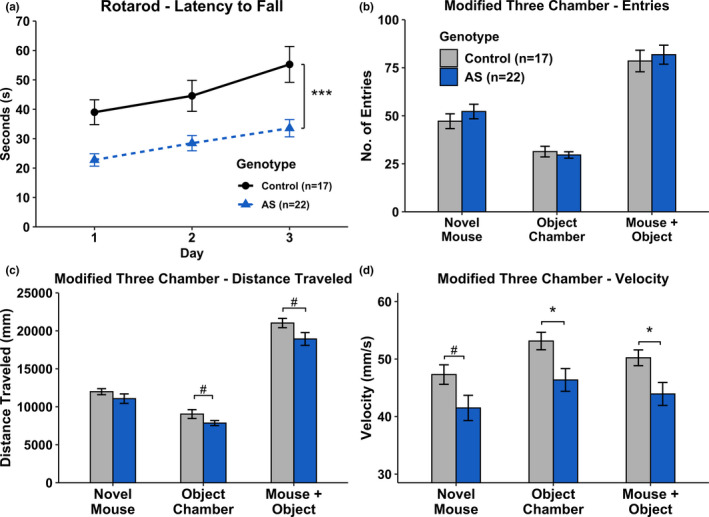
Motor analyses. (a) *Ube3a^m‐/p+^* mice display poor sensorimotor ability but typical motor learning as observed on the rotarod task. AS mice spent significantly less time on the accelerating rotating cylinder when compared to Control mice. Further motor‐related behaviors were assessed used the Modified Three‐Chamber Social Task (b–d). (b) No significant differences between Control and AS mice on number of entries into each chamber on the Modified Three‐Chamber Social Task. (c) AS mice displayed decreased distance travelled and (d) decreased velocity in the Modified Three‐Chamber Social task. *#p* < .10; *p < .05; ***p < .001

Using a 2 × 3 repeated measures ANOVA, we found a main effect of Genotype [*F*(1, 37) = 14.37, *p* < .001] on latency to fall, with AS mice remaining for significantly shorter intervals on the Rotarod indicating poor motor coordination (Figure 2a). Genotype did not interact with Day, however, and both groups showed significant Day effects when assessed separately [WT: *F*(2, 32) = 9.500, *p* = .001; AS: *F*(2, 42) = 7.320, *p* = .002].

Various motor‐related measurements within the Modified Three‐Chamber Social Task were assessed using a one‐way ANOVA. There was no main effect of Genotype when analyzing number of entries into each chamber [Novel Mouse: *F*(1, 37) = 0.851, *p* > .05; Novel Object: *F*(1, 37) = 0.327, *p* > .05; Mouse + Object Chambers: *F*(1, 37) = 0.193, *p* > .05] (Figure 2b). AS mice did show a trend toward decreased distance travelled in the Novel Object Chamber and the combination of the Novel Mouse and Novel Object Chambers, but not the Novel Mouse Chamber alone [Novel Mouse: *F*(1, 37) = 1.340, *p* > .05; Novel Object: *F*(1, 37) = 3.496, *p* < .10; Mouse + Object Chambers: *F*(1, 37) = 3.634, *p* < .10] (Figure 2c). Additionally, AS mice were trending toward a decreased velocity in the Novel Mouse chamber and were significantly slower than Control mice in the Novel Object Chamber and the combination of both chambers [Novel Mouse: *F*(1, 37) = 3.971, *p* < .10; Novel Object: *F*(1, 37) = 6.700, *p* < .05; Mouse + Object Chambers: *F*(1, 37) = 5.972, *p* < .05] (Figure 2d). Additionally, a significant correlation was found between Rotarod performance and Three‐Chamber velocity (Pearson's correlation: *R* = .445, *p* = .038), further supporting a motor deficit in AS mice. This correlation was not seen in Control mice (*p* > .05). Analysis of motor behaviors within the Modified Three‐Chamber Social Task supports the motor impairments seen in AS mice on the Rotarod task.

### 3.2 Modified three‐chamber social task

**Figure 3:**
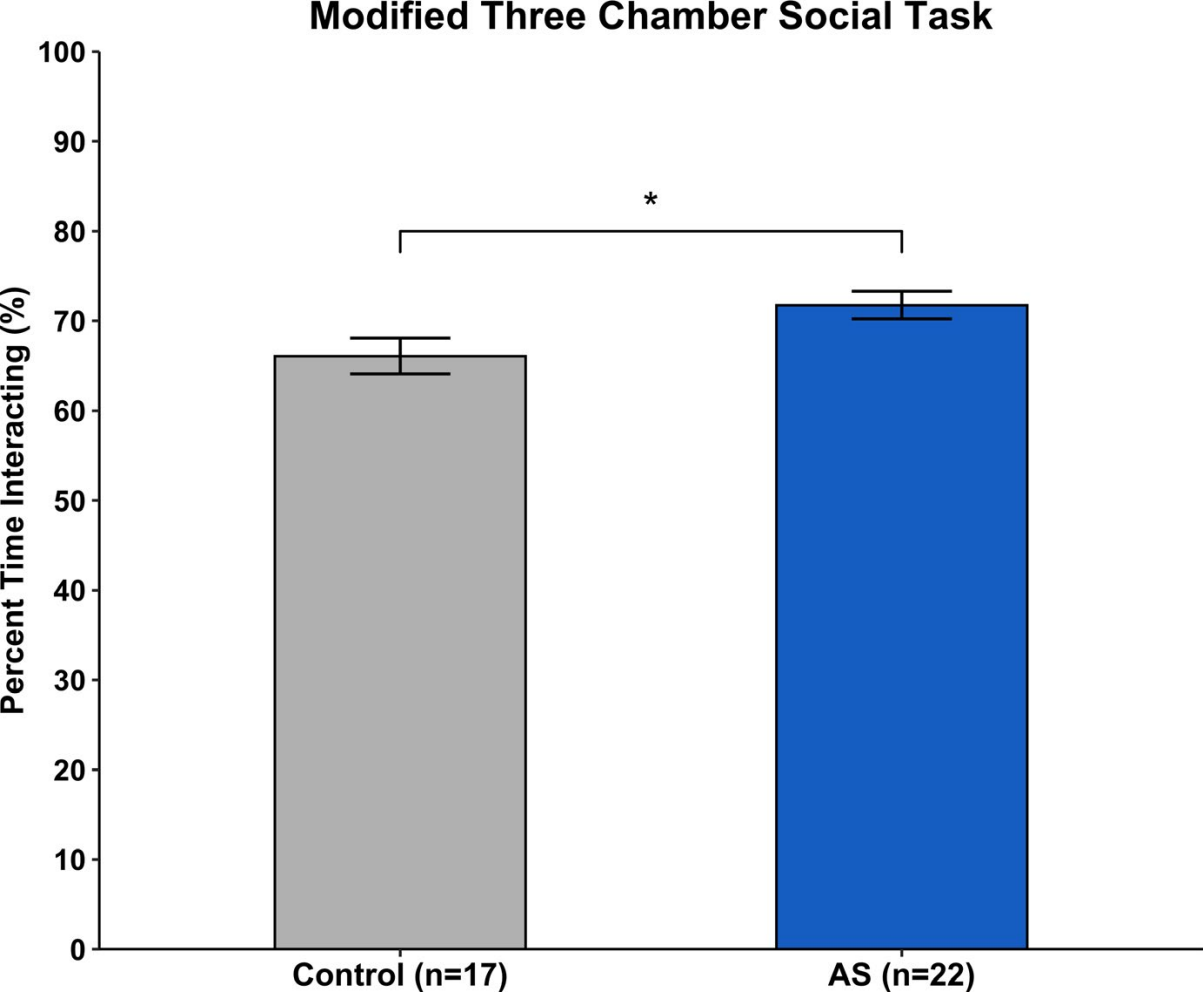
Social preference. *Ube3a^m−/p+^* mice spend significantly more time interacting with a novel conspecific mouse as compared to Control mice. *p < .05

Results from the Modified Three‐Chamber Social task showed a main effect of Genotype [*F*(1, 37) = 5.219, *p* < .05] on percent time interacting with conspecific. AS mice spent significantly more time interacting with the conspecific mouse, indicating atypical social behavior (Figure 3).

### 3.3 Ultrasonic Vocalizations

**Figure 4:**
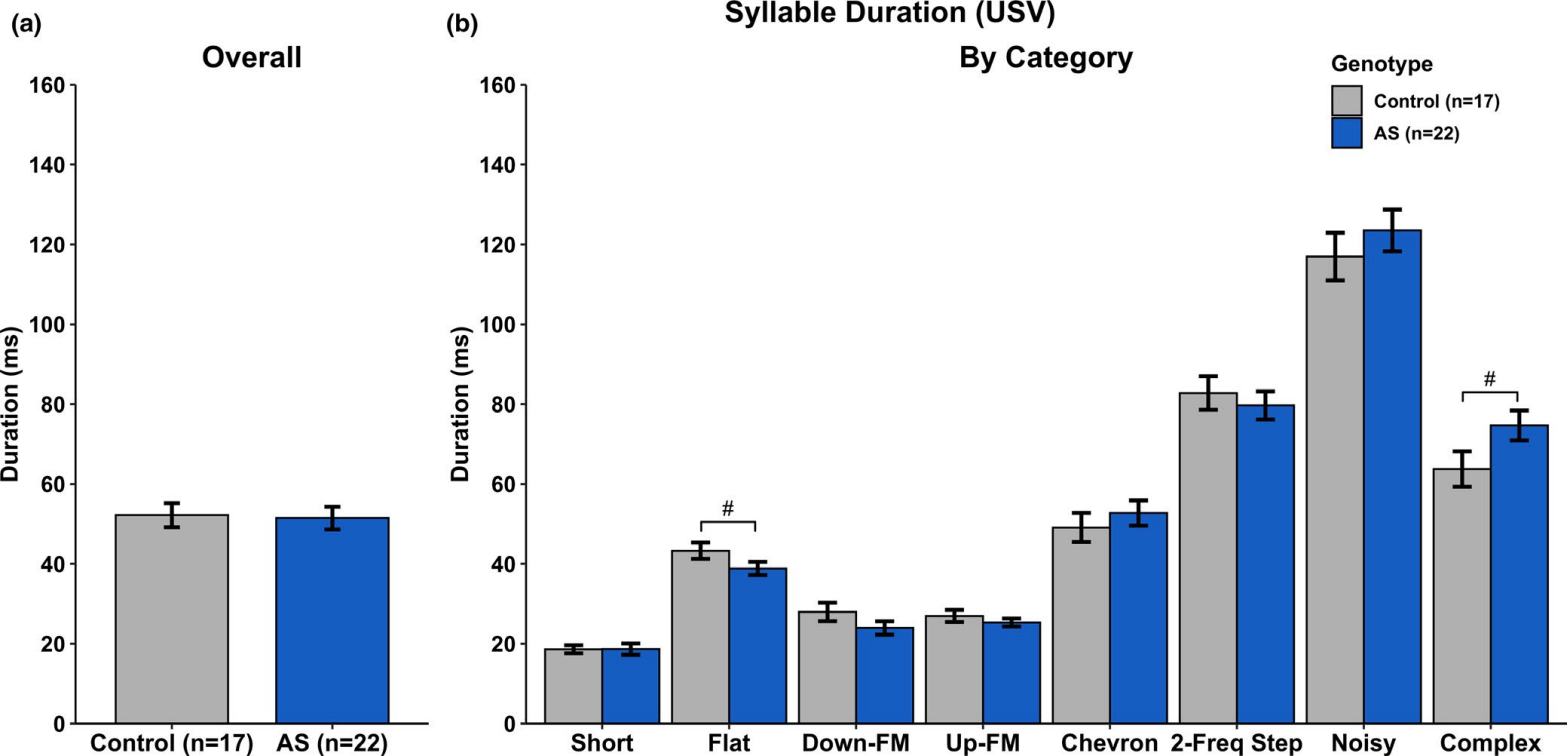
Ultrasonic Vocalizations—Syllable Duration. a) There was no main effect of Genotype on syllable duration when collapsed by category. b) *Ube3a^m−/p+^* mice produced shorter Flat syllables when compared to Control mice. All other syllable categories did not show a main effect of Genotype. ^#^ *p* < .10

**Figure 5:**
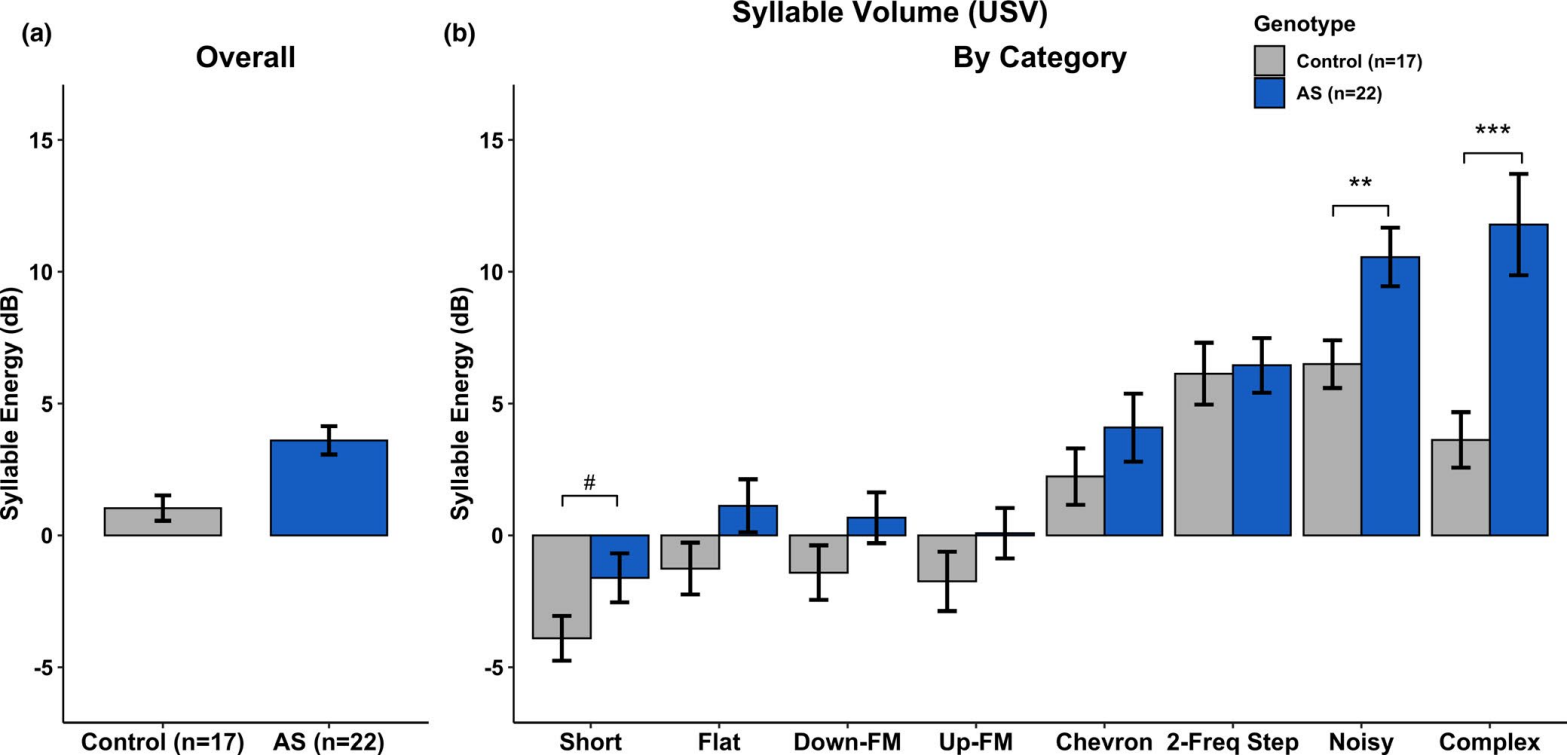
Ultrasonic Vocalizations—Syllable Volume. a) There was no main effect of Genotype on syllable volume when collapsed by category. b) *Ube3a^m‐/p+^* mice produced louder Short, Noisy, and Complex syllables when compared to Control mice. All other syllable categories did not show a main effect of Genotype. ^#^ *p* < .10; **p < .01; *** *p* < .001

**Figure 6:**
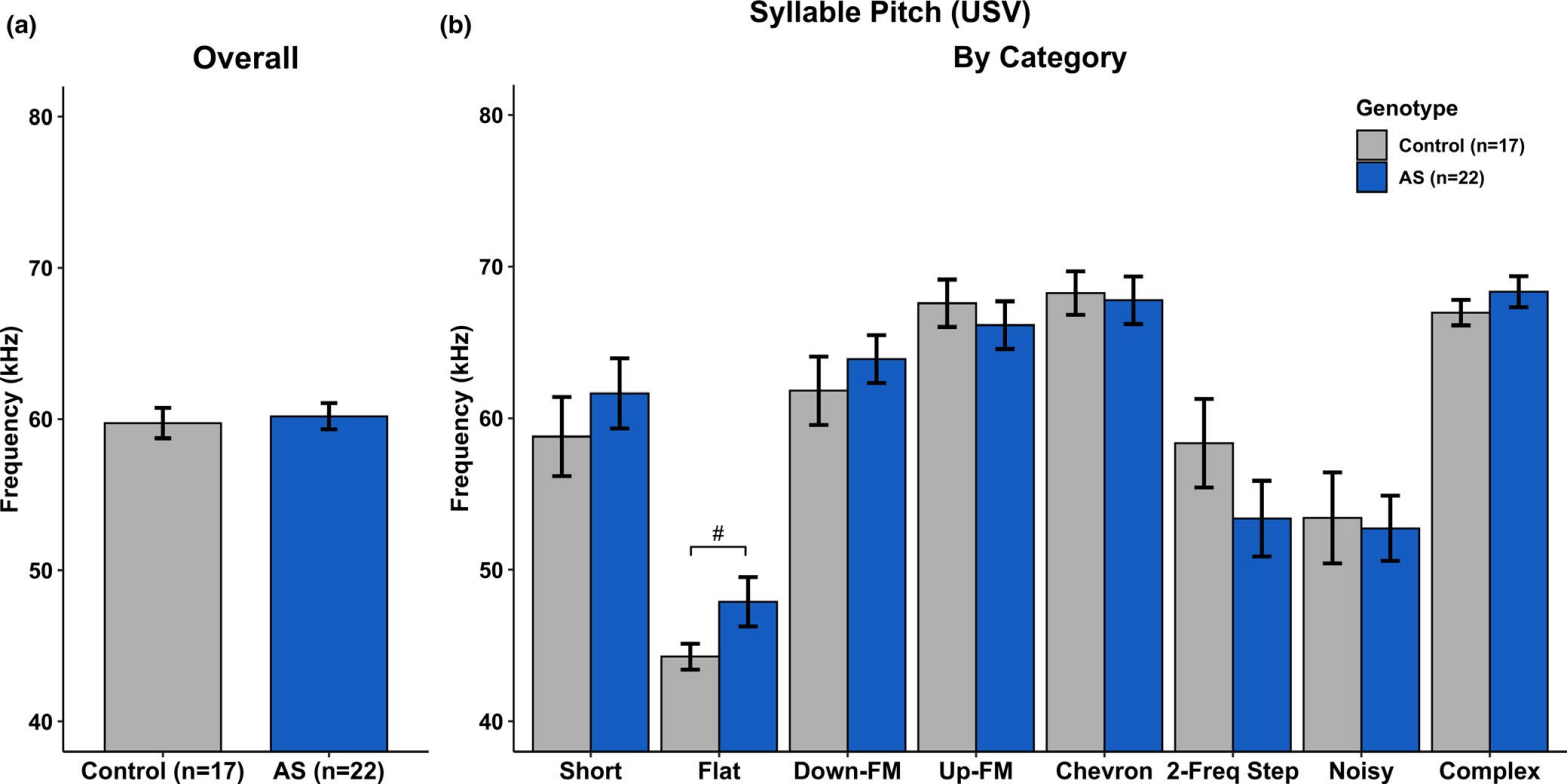
Ultrasonic Vocalizations—Syllable Pitch. a) There was no main effect of Genotype on syllable volume when collapsed by category. b) *Ube3a^m‐/p+^* mice produced higher pitched Flat syllables when compared to Control mice. All other syllable categories did not show a main effect of Genotype. ^#^ *p* < .10

**Figure 7:**
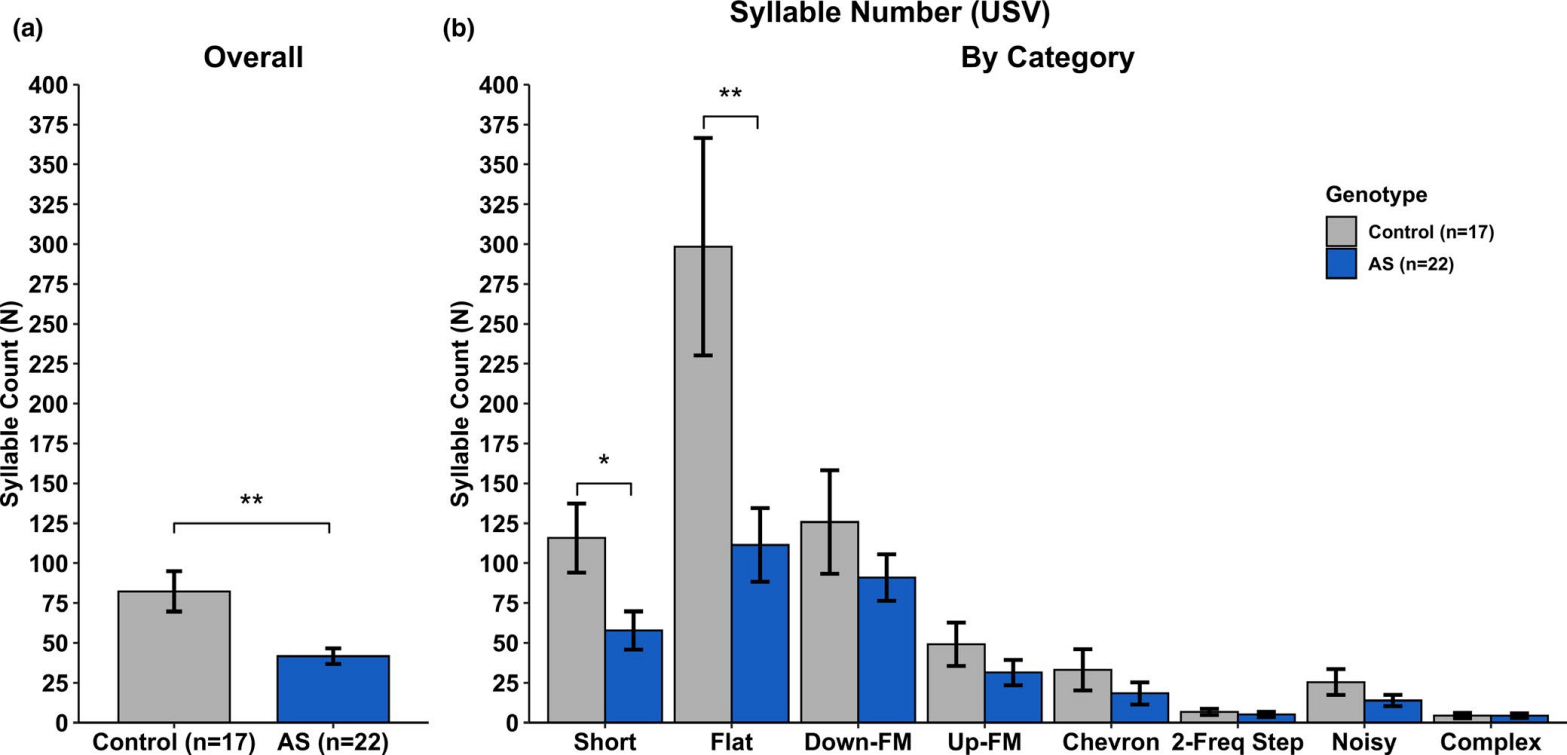
Ultrasonic Vocalizations—Syllable Number. a) *Ube3a^m−/p+^* mice produce fewer vocalizations when compared to Control mice. b) *Ube3a^m−/p+^* mice produced less Short and Flat syllables when compared to Control mice. All other syllable categories did not show a main effect of Genotype. *p < .05; **p < .01

**Figure 8:**
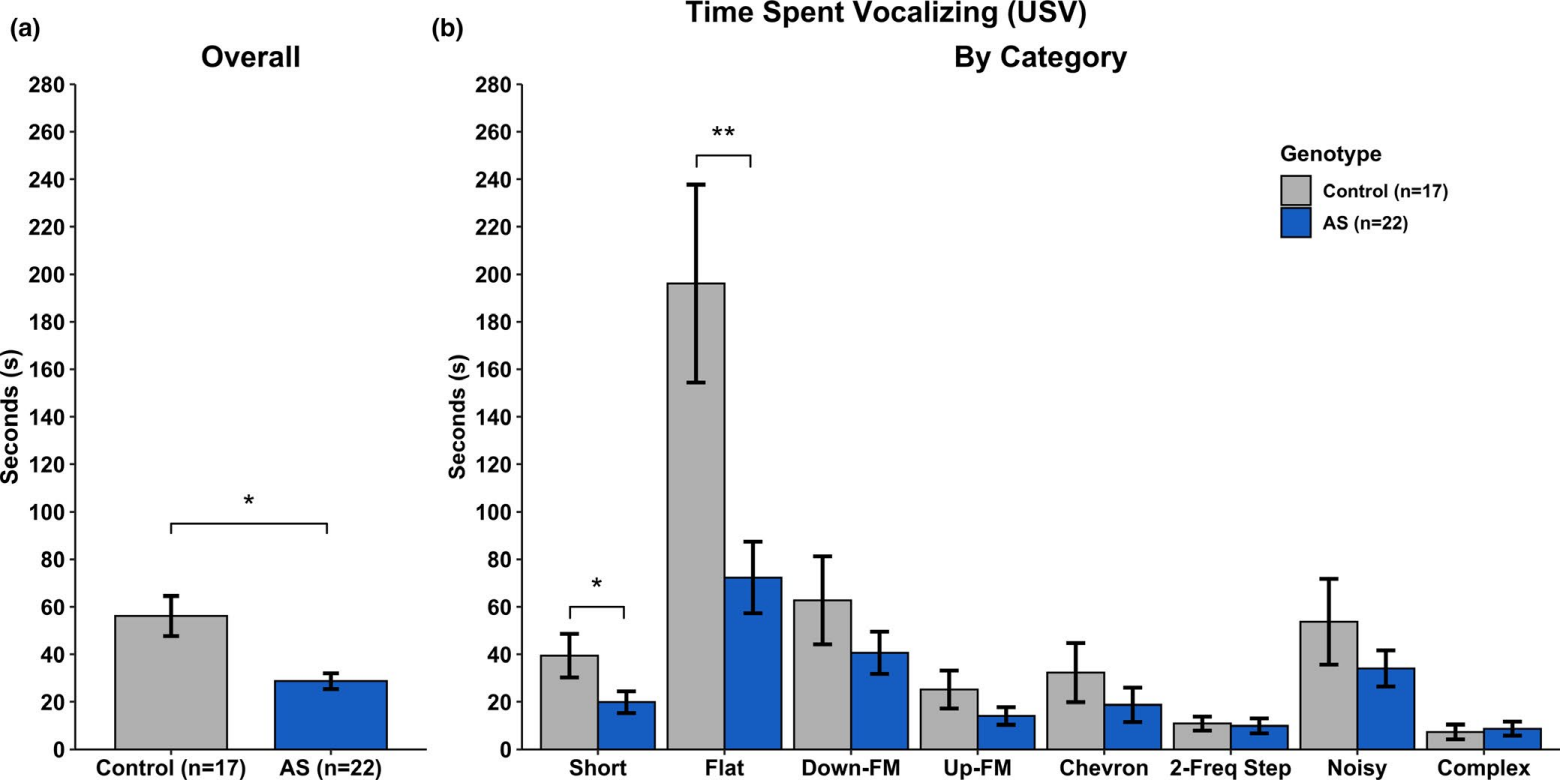
Ultrasonic Vocalizations—Time Spent Vocalizing. a) *Ube3a^m−/p+^* mice spent less time vocalizing when compared to Control mice. b) *Ube3a^m−/p+^* mice spent less time producing Short and Flat syllables when compared to Control mice. All other syllable categories did not show a main effect of Genotype. *p < .05; **p < .01

USV measures included syllable duration, syllable volume, syllable pitch, the total number of syllables produced, and total time spent vocalizing were analyzed. A complete breakdown of the statistical analyses can be found in Tables 1, 2, 3, 4, and 5. We found no statistical differences between groups in overall Syllable Duration (Figure 4), overall Syllable Volume (Figure 5), and overall Syllable Pitch (Figure 6). However, AS mice produced statistically fewer syllables (Figure 7) and spend less time vocalizing (Figure 8) than Control mice. However, we did find that AS mice trended to produce Flat vocalizations that were longer in duration [*F*(1, 37) = 2.938, *p* < .10], higher in pitch [*F*(1, 37) = 3.259, *p* < .10], and fewer in number [*F*(1, 37) = 3.259, *p* < .10]. They also vocalized for a significantly shorter amount of time than Control mice [*F*(1, 37) = 9.430, *p* < .05]. Additionally, AS mice produced Short vocalizations that were louder in volume [*F*(1, 36) = 3.164, *p* < .10], fewer in number [*F*(1, 37) = 6.154, *p* < .05] and vocalized for a shorter amount of time than Control mice [*F*(1, 36) = 4.066, *p* = .05]. Furthermore, AS mice produced louder Noisy and Complex calls when compared to Control mice [Noisy: *F*(1, 32) = 7.844, *p* < .05; Complex: *F*(1, 26) = 12.694, *p* = .001].

**Table 1:** Statistics—syllable duration

Category	*df*	*F*	*p*
Overall	1, 37	0.209	.650
Short	1, 36	0.001	.971
*Flat*	*1, 37*	*2.938*	*^#^.095*
Down‐FM	1, 36	2.073	.159
Up‐FM	1, 37	0.862	.359
Chevron	1, 34	0.576	.453
2‐Frequency Step	1, 28	0.324	.574
Noisy	1, 32	0.681	.415
*Complex*	*1, 26*	*3.636*	*^#^.068*

Note

Statistics illustrating main effect of Genotype for Syllable Duration collapsed by Category (Overall) and by the 8 observed categories. Italics indicate significance: #*p* < .10; *p < .5; **p < .01; ***p < .001. *df* reflects only the subjects generating at least one of the syllable types (not all subjects produced all vocalization types).

**Table 2:** Statistics—syllable volume

Category	*df*	*F*	*p*
Overall	1, 37	1.901	.176
*Short*	*1, 36*	*3.164*	*^#^.084*
Flat	1, 37	2.759	.105
Down‐FM	1, 36	2.153	.151
Up‐FM	1, 37	1.536	.223
Chevron	1, 34	1.153	.290
2‐Frequency Step	1, 28	0.041	.842
*Noisy*	*1, 32*	*7.844*	**.009
*Complex*	*1, 26*	*12.694*	***.001

Note

Statistics illustrating main effect of Genotype for Syllable Volume collapsed by Category (Overall) and by the 8 observed categories. Italics indicate significance: #*p* < .10; *p < .5; **p < .01; ***p < .001. *df* reflects only the subjects generating at least one of the syllable types (not all subjects produced all vocalization types).

**Table 3:** Syllable pitch

Call type	*df*	*F*	*p*
Overall	1, 37	0.002	.964
Short	1, 36	0.666	.420
*Flat*	*1, 37*	*3.259*	*^#^.079*
Down‐FM	1, 36	0.607	.441
Up‐FM	1, 37	0.409	.527
Chevron	1, 34	0.048	.827
2‐Frequency Step	1, 28	1.700	.203
Noisy	1, 32	0.035	.852
Complex	1, 26	1.066	.311

Note

Statistics illustrating main effect of Genotype for Syllable Pitch collapsed by Category (Overall) and by the 8 observed categories. Italics indicate significance: #*p* < .10; *p < .5; **p < .01; ***p < .001. *df* reflects only the subjects generating at least one of the syllable types (not all subjects produced all vocalization types).

**Table 4:** Statistics—syllable number

Call type	*df*	*F*	*p*
*Overall*	*1, 37*	*7.497*	**.009
*Short*	*1, 36*	*6.154*	*.018
*Flat*	*1, 37*	*8.213*	**.007
Down‐FM	1, 36	1.120	.297
Up‐FM	1, 37	1.397	.245
Chevron	1, 34	1.134	.294
2‐Frequency Step	1, 28	0.387	.538
Noisy	1, 32	2.018	.164
Complex	1, 26	0.000	.988

Note

Statistics illustrating main effect of Genotype for Syllable Number collapsed by Category (Overall) and by the 8 observed categories. Italics indicate significance: #*p* < .10; *p < .5; **p < .01; ***p < .001. *df* reflects only the subjects generating at least one of the syllable types (not all subjects produced all vocalization types).

**Table 5:** Statistics—time spent vocalizing

Call type	*df*	*F*	*p*
*Overall*	*1, 37*	*6.546*	*.015
*Short*	*1, 36*	*4.066*	*.051
*Flat*	*1, 37*	*9.430*	**.004
Down‐FM	1, 36	1.310	.260
Up‐FM	1, 37	1.864	.180
Chevron	1, 34	0.968	.332
2‐Frequency Step	1, 28	0.051	.822
Noisy	1, 32	1.098	.303
Complex	1, 26	0.105	.748

Note

Statistics illustrating main effect of Genotype for Time Spent Vocalizing collapsed by Category (Overall) and by the 8 observed categories. Italics indicate significance: #*p* < .10; *p < .5; **p < .01; ***p < .001. *df* reflects only the subjects generating at least one of the syllable types (not all subjects produced all vocalization types).

### 3.4 Normal Single Tone

**Figure 9:**
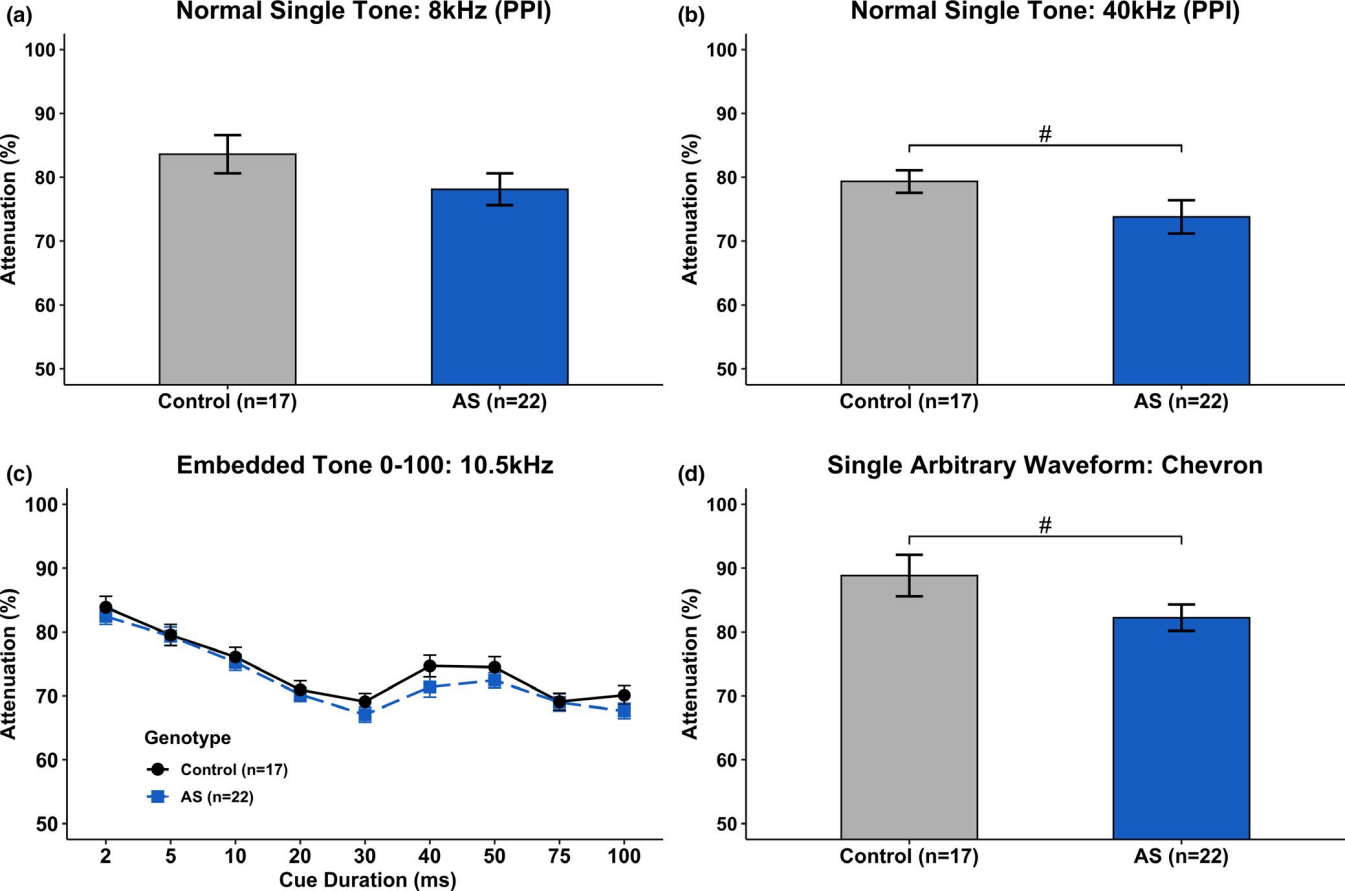
Rapid auditory processing ability. a) *Ube3a^m−/p+^* mice did not show any impairments or enhancements on Normal Single Tone (NST) at 8kHz but did show a marginal enhancement at 40kHz (b). c) When collapsed over 5 days, *Ube3a^m−/p+^* mice and Control mice performed similarly on Embedded Tone 0–100 at 10.5 kHz. D) *Ube3a^m−/p+^* mice showed a marginal enhancement on Single Arbitrary Waveform (SAW). #*p* < .10. *Lower attenuation scores indicate better performance*

Analysis of NST 8kHz did not reveal a main effect of Genotype [*F*(1, 37) = 2.016, *p* > .05]. However, AS mice trended better than Control mice on NST 40kHz [*F*(1, 37) = 2.752, *p* < .10], suggesting a modest cue detection ability at ultrasonic frequencies.(Figure 9a & 9b).

### 3.5 Embedded Tone 100

EBT 100 was analyzed using NST 8kHz as a covariate to account for individual differences. A repeated measures ANOVA did not reveal a main effect of Genotype [*F*(1, 36) = 0.184, *p* > .05] (Figure 9c). Thus, *Ube3a* AS and Control were statistically similar in their ability to detect a rapidly presented auditory cue that varied in duration.

### 3.6 Single Arbitrary Waveform

A univariate ANOVA revealed that AS mice trended better than Control mice on SAW [*F*(1, 37) = 3.207, *p* < .10], suggesting AS mice have a modest enhancement when detecting and responding to ultrasonic vocalizations, specifically a C57 wild‐type Chevron (Figure 9d).

### 3.7 Path modeling, USV Production

**Figure 10:**
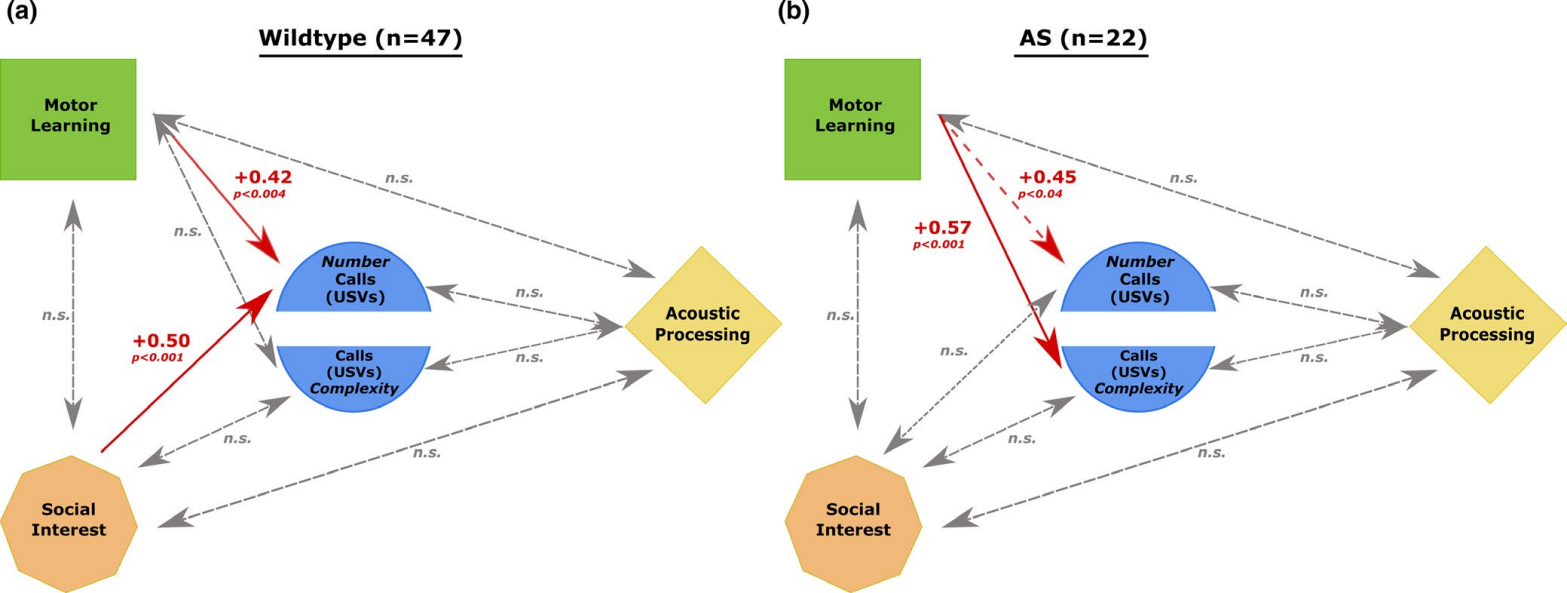
Path Modeling Analyses. A) Path modeling analysis for all wild‐type (WT) mice (*n* = 47). Results show significant positive linear bivariate correlations between Motor Learning and USV production & Social Preference and USV production. B) Path modeling analysis for *Ube3a^m−/p+^* (AS) mice (*n* = 22). Results showed a marginal positive correlation between Motor Learning scores and USV production that did not survive α‐correction. A strong correlation between Motor Learning and USV complexity was found. Dashed gray arrows indicate nonsignificant correlations (n.s.); dashed red arrows indicate trending correlations (α = 0.013, adjusted for multiple comparisons); solid red arrows indicate strong positive correlations (*p* < .013)

To develop a baseline for comparison, we analyzed relationships between behavioral scores obtained from adult wild‐type C57 mice across *four* transgenic behavioral studies, including the wildtypes from the current study (*n* = 47; 17f/30m). *See* Naveh, 2019 *,* Rendall et al., 2016 *&* Rendall et al., 2017 *for experimental details for each study*. Comparable tasks, conditions, and measures were used across studies. The use of aggregate data from 4 studies increased the number of wild‐type subjects used and therefore enhanced the reliability of the correlational analyses. Individual mean scores were standardized to *Z*‐scores within Task/Study, for Motor Learning, Social Preference, and Auditory Processing. These scores showed no multicollinearity (i.e., did not correlate with each other), and auditory processing scores did not relate to USV production. However, Motor and Social scores *did* associate with overall USV production, showing positive linear bivariate correlations to Motor (+0.42, *p < *.004) and Social (+0.5, *p < *.001) scores (significant with α corrected to 0.013 for multiple tests) (Figure 10a). We also used linear multiple regression to evaluate combined influence(s) on USV production. A model using both Motor Learning and Social Preference scores most effectively predicted USV output in Control mice, accounting for 36% of the variance in USV production (*F* = 12.0 (*df*, 2, 44), *p < *.005, *R* ^2^ = .36; Motor Learning, *t* = 2.74, *p < *.01, Social Preference, *t* = 3.71, *p < *.005). This confirmed that both Motor and Social indices positively and significantly relate to USV production in typically developing adult C57 mice.

Importantly, the subset of data from wild‐type females only (*n* = 17) was separately re‐assessed, since the mice in the current study were all female. Analyses confirmed that Auditory Processing scores did not relate to predict USV production in wild‐type females, whereas significant positive linear bivariate correlations were again seen between Motor/USV (+0.53) and Social Preference/USV (+0.45; *n* = 17). Similar results were seen for the remaining cohort of wild‐type males (Motor/USV = +0.38; Social/USV = +0.53; *n* = 30). Independent confirmation of the same pattern in both sexes supports the viability of the statistical model (data not shown). These results follow a similar pattern to analyses that contained wild‐type mice from the combination of four studies (see above); however, the *n* = 17 control females from this study displayed less robust findings.

Next, we performed the same analyses for the AS model mice only (*n* = 22 *Ube3a* AS female littermates of control females from above). Again, Motor, Social, and Auditory indices showed no co‐linearity and auditory processing scores did not relate to USV production. Motor Learning scores continued to show a trend toward positive correlation with USV production that did not survive α‐correction (+0.45, *p* < .04). However, a correlation between Motor and the specific production specifically of *complex* USV calls (chevron, 2‐frequency step, Complex and Noisy only; Table 5), showed a strong and positive Motor/USV correlation of +0.57 that survived correction (*p* < .001). Similar analysis of Social indices revealed no correlations with either total USV production, or complex USV production (n.s.) (Figure 10b) Our interpretation of a motor contribution to altered communication in the AS mouse model is supported by significant correlations between motor indices and USV production (syllable number and time spent vocalizing)—correlations that were not seen in Control mice. Additional analyses were conducted using mean velocity in the Modified Three‐Chamber task in place of Rotarod performance. Mean velocity did not reach significance in correlating with USV production (i.e., time spent vocalizing or number of vocalizations produced) in AS mice (*p* > .05). The nonsignificant mean velocity—USV production correlation reported here may be contaminated by aberrant social preferences—future studies plan to assess additional motor measures during the USV recording process to replicate and confirm the motor‐USV correlation in AS mice.

## 4 DISCUSSION

The current study was designed to further explore communicative anomalies associated with AS using an established mouse model. We replicated evidence that disruptions to maternally expressed *Ube3a* are associated with impaired motor coordination and learning, and atypically enhanced social behaviors. Novel results show enhanced *receptive auditory processing* ability in baseline prepulse inhibition and social‐communicative stimuli. We also report significantly *decreased* ultrasonic vocalization production, consistent with an AS phenotype. Finally, statistical analyses reveal that motor deficits—but *not* social or acoustic processing anomalies—are strongly correlated to atypical vocal output in the AS model, suggesting that neurologic anomalies in the motor system are the primary contributor to communicative impairments in the mouse model of AS.

### 4.1 AS mice display AS‐like phenotypes

Our results validate the use of an *Ube3a* AS mouse to study the behavioral consequences of maternal‐*UBE3A* disruption, as we report phenotypes consistent with individuals with AS. Specifically, AS mice displayed motor impairments on the Rotarod task and within the Modified Three‐Chamber Social Task—these results indicate that AS model mice show motor learning, with a learning‐curve comparable to Control mice, but also demonstrate a baseline motor coordination deficit that persists across learning. These results are important in that they suggest AS model mice can attain motor performance levels of controls with additional training. AS mice also displayed atypical social behavior in a Modified Three‐Chamber Social Task—AS mice spent more time interacting with the novel, caged mouse. Previous work behaviorally characterizing *Ube3a* mouse models report impairments on the Rotarod task (Bruinsma et al., 2015; Heck et al., 2008; Huang et al., 2013; Sonzogni et al., 2018), as well as increased social behavior. Using a slightly different protocol, Stoppel and Anderson (2017) reported that female mice with a maternally inherited *Ube3a* deletion spent prolonged periods of time with a novel social stimulus in the Three‐Chamber Social task, exhibiting a hyper‐social phenotype. These motor‐ and social‐related phenotypes parallel human clinical findings—individuals with AS experience movement impairments (i.e., an ataxic gait and tremulous limb movements) and altered social behavior (i.e., frequent laugher/happy demeanor and an easily excitable personality) (Williams et al., 1995; Williams et al., 2006). The combination of the Rotarod an Modified Three‐Chamber Social Task results raises an interesting problem—are AS mice traveling less distance and moving slower due to an overall motor impairment or due to a social fixation on the novel mouse? More fine‐tuned rodent behavioral tasks are necessary to fully explore this relationship and the general relationship between maternal *UBE3A* disruption and motor‐ and social‐related alteration.

### 4.2 Auditory processing enhancements in AS mice

We evaluated AS mice on additional tasks relevant to human language development and acquisition to better understand how *UBE3A* impairs language ability. Our study is the first to show that AS mice exhibit *enhanced* cue detection and prepulse inhibition on auditory processing tasks (i.e., NST 40kHz and SAW). This finding is particularly relevant because early auditory processing scores can accurately predict language outcomes (e.g., vocabulary and other language scores) from human infants (Benasich *et al.,* 2002). Low‐level perceptual discrimination enhancements have been reported in other neurodevelopmental disorders, including ASD (Bertone et al., 2005; Mottron et al., 2006; Plaisted et al., 2003) and may contribute to the language‐related impairments seen in the disorder. In particular, it has been suggested that *enhancements* in auditory processing ability can also contribute to poor language outcomes (Eigsti & Fein, 2013). These findings may also hold for AS, but further research is necessary. Research on this topic has been hindered by the frequent lack of oral language in AS, which restricts the use of common language‐based outcome measures, although novel studies with mosaic AS populations that show intact communication may provide new insights (Carson et al., 2019; Eigsti and Chamberlain, personal communication). Further mouse studies can also interrogate the spectrotemporal parameters of any acoustic processing anomalies, and neural histology is needed to quantify possible cellular and structural aspects of auditory‐related brain structures in AS model mice. Indeed, we have previously reported abnormalities in thalamic nuclei and various white matter structures in related ASD model mice (Rendall et al., 2017). This approach could ultimately inform targeted interventions to enhance language development in affected individuals.

### 4.3 Alterations in AS mice ultrasonic vocalizations

To our knowledge, this study provides the first thorough characterization of adult *Ube3a* AS mouse ultrasonic vocalizations. We found that AS mice produced fewer calls and vocalized for a shorter amount of time than Control mice. These novel results contrast conflicting literature reports on USV patters in AS models suggesting a reduction in calls (Mandel‐Brehm et al., 2015; Stoppel & Anderson, 2017). Ultrasonic vocalizations in juveniles reflect a pup's ability to seek maternal care by generating distress calls after being separated from their mother. This paradigm has frequently been used to show reduced vocalizations in ASD mouse models (Fischer & Hammerschmidt, 2011; Scattoni et al., 2009). Prior studies of the AS mouse model using this task include work from Mandel‐Brehm et al., (2015), who showed that AS mouse pups produce more USVs at P13 through P15 when compared to wild‐type mice. This result was originally interpreted as a general enhancement in vocal communication, which was puzzling since humans with AS show limited or absent vocal speech communication. However, a more fine‐grained examination of those data reveals a developmental “delay” in peak distress call production in AS mouse pups, such that distress call production peaks at 15–17 days in the AS pups compared with 5–7 days in the wild‐type pups. This suggests a maturational lag rather than an overall increase in vocalizations in AS pups, a pattern that may be analogous to the developmental delay of ~4 days in peak USV distress calls in Down‐syndrome model mouse pups (Holtzman et al., 1996).

A second potentially conflicting finding was reported by Stoppel and Anderson (2017) who described *increased* USVs in adult female AS mice. However, this study was conducted using a different background strain than our experiments (FVB), a different recording protocol, and most importantly, a different housing protocol. In particular, these investigators showed evidence that group‐housed female AS model mice produced significantly more vocalizations than WTs, while single‐housed AS model mice showed a trend to the opposite pattern (similar to our results). Our subjects were single‐housed following weaning at P21. Further research is needed to fully explore the effect of group versus single‐housing on USV production, but our results are the first to show that single‐housed, adult female AS mice produce significantly *fewer* USVs and vocalize for shorter amounts of time when paired with a novel conspecific.

### 4.4 Genetic effects on domain‐specific pathways that alter communication

A major barrier in the use of animal models to address language and communication‐based neurodevelopmental disorders has centered on the disputed validity of rodent communication systems as useful models of higher‐order human language systems (e.g., Okabi *et al.,* 2019). Many arguments in particular have been advanced that mouse calls are “reflexive” or brainstem‐mediated responses, with little element of voluntary control, thus undermining modeling efforts. New research, however, shows that mice indeed possess rudimentary circuitry that includes orofacial/laryngeal motor cortex subserving voluntary regulation of USV generation (Okobi *et al.,* 2019). Moreover, orthogonal circuitry in the mouse appears to regulate the socio‐emotional components of their USV production (periaqueductal gray; Tschida *et al.,* 2019). And, finally, left‐hemisphere specialization for the *processing* of USVs (relative to general spectrotemporal acoustic information) has been documented in mouse auditory cortex (Levy *et al.,* 2019). These findings bolster the value of mouse models of communication to tap precursors to the multiple systems subserving language and communication in the human brain. As such, it becomes critically important to evaluate which circuits in particular appear to be affected in neurodevelopmental disorders such as AS. These studies are difficult to perform in humans, in part because the complex and profound interactive influences of emergent language in humans make the dissociation of domain‐specific systems quite difficult. This is because, for example, anomalies in processing input that lead to speech delays may have subsequent impact on social verbal interactions, as well as altering educational experiences and development of higher language skills, making dissociation of “causal deficits” in communicative processes all but impossible at older ages. Though mouse studies also suffer confounds from developmental experience, the simplicity of communicative systems coupled with a highly consistent and controlled environment, and the power of genetic manipulation, mitigate these confounds to some degree.

To directly address the important question of which circuits are affected in the mouse AS model, we assessed the correlations within subjects between scores on motor learning tasks, acoustic processing tasks, social interaction tasks, and USV production. Rather than finding a general intercorrelation of all measures, reflecting broad within‐subjects symptom‐severity effects, results indicated that motor indices correlated strongly with anomalous USV output. This effect was magnified when only complex USV call production was considered, perhaps due to greater precision in oral‐motor control required for more complex vocalizations. Neither social nor acoustic processing measures appeared to relate to USV output in the AS model mice, despite significant AS model group differences on both of those measures. Notably, results from a *Shank3b* ASD mouse model (unpublished data) follow a distinct pattern, with a more robust statistical contribution to USV production from Social rather than Motor indices. This suggests that the role of motor processes in communicative impairments may be a specific to Angelman Syndrome.

Interestingly, we observed a reduction in vocalization production (i.e., number of vocalizations produced and time spent vocalizing) and atypical social behavior (i.e., increased time spent with novel mouse) in AS mice—two results that seem contradictory—one might expect increased social behavior and increased vocalization production. However, these results are consistent with symptoms of AS (i.e., heightened sociability despite communicative deficits). If motor impairments are driving reductions in vocalizations, perhaps AS mice are compensating via other various methods of communication (i.e., increased sniffing). Future work requires a detailed analysis of how AS mice interact when measuring ultrasonic vocalizations.

## 5 CONCLUSION

The current findings replicate prior evidence of an AS‐like behavioral phenotype in *Ube3a^m−/p+^* mice and provide novel findings into how *Ube3a* mutations in mice affect rapid auditory processing ability and ultrasonic vocalization production. Evidence from AS model mice suggests a strong motor contribution to communicative impairments. Coupled with evidence that AS model mice show motor learning at rates comparable to control mice (despite baseline deficits that persist), combined findings offer substantial promise for the success of motor interventions and therapies in improving communicative performance of individuals with AS. Furthermore, findings suggest that language‐rescue efforts in AS populations should focus on treatments and timeframes that can *rescue motor ability*. Ongoing studies will further assess the correlation between motor activity and ultrasonic vocalization production—future studies will include more fine‐grained measures during ultrasonic vocalization recordings to evaluate the direct motor‐ and social interaction between subjects.

## CONFLICT OF INTEREST

The authors have no conflicts of interest to declare.

## AUTHOR CONTRIBUTIONS

P.A.P. was responsible for generating subjects (i.e., breeding, general animal husbandry, and genotyping), conducting and overseeing behavioral experiments, analyzing the data, and drafting the manuscript. S.J.C played a crucial role in genotyping subjects and experimental design. S.J.C and I.M.E were responsible for planning and drafting the manuscript. R.H.F contributed valuable insight into experimental design, data interpretation, and manuscript prep/drafting. All authors reviewed and agreed with the content of the manuscript.

### Peer Review

The peer review history for this article is available at https://publons.com/publon/10.1002/brb3.1937.

## Data Availability

Data that support these findings can be made available upon reasonable request.
